# Fecal Calprotectin and Eosinophil-Derived Neurotoxin in Children with Non-IgE-Mediated Cow’s Milk Protein Allergy

**DOI:** 10.3390/jcm10081595

**Published:** 2021-04-09

**Authors:** María Roca, Ester Donat, Ana Rodriguez Varela, Eva Carvajal, Francisco Cano, Ana Armisen, Helena Ekoff, Antonio José Cañada-Martínez, Niclas Rydell, Carmen Ribes-Koninckx

**Affiliations:** 1 Celiac Disease and Digestive Immunopathology Unit, Instituto de Investigación Sanitaria La Fe, 46026 Valencia, Spain; donat_est@gva.es (E.D.); ribes_car@gva.es (C.R.-K.); 2 Pediatric Gastrohepatology Unit, Hospital Universitario y Politécnico La Fe, 46026 Valencia, Spain; 3 Pediatrics, Primary Health Care Center of Betera, 46117 Valencia, Spain; ana.ro.va@hotmail.com (A.R.V.); koyaanis5@hotmail.es (F.C.); ana.armisen@gmail.com (A.A.); 4 Department of Paediatrics, Hospital Casa de Salud, 46021 Valencia, Spain; eva.carvajal2@gmail.com; 5 Thermo Fisher Scientific, 754 50 Uppsala, Sweden; helena.ekoff@thermofisher.com (H.E.); niclas.rydell@thermofisher.com (N.R.); 6 Biostatistics Unit, Instituto de Investigación Sanitaria La Fe, 46026 Valencia, Spain; bioestadistica@iislafe.es

**Keywords:** fecal calprotectin, eosinophil-derived neurotoxin, non-IgE-mediated cow’s milk protein allergy

## Abstract

Our aim is to assess the efficacy of fecal calprotectin (fCP) and fecal eosinophil-derived neurotoxin (fEDN) as diagnostic markers of cow’s milk protein allergy (CMPA) and for monitoring the infants’ response to a non-IgE mediated cow’s milk protein (CMP)-free diet. We prospectively recruited infants aged 0 to 9 months. Stool samples were taken from 30 infants with CMPA, 19 with mild functional gastrointestinal disorders, 28 healthy infants, and 28 children who presented mild infections. Despite the fact that levels of fCP and fEDN in CMPA infants were higher than in healthy infants at month 0, differences for both parameters did not reach statistical significance (*p-*value 0.119 and 0.506). After 1 month of an elimination diet, no statistically significant differences in fCP with basal levels were found (*p-*values 0.184) in the CMPA group. We found a high variability in the fCP and fEDN levels of young infants, and discrepancies in individual behavior of these markers after a CMP-free diet was started. It seems that neither fCP nor fEDN levels are helpful to discriminate between healthy infants and those with signs or symptoms related to non-IgE-mediated CMPA. Additionally, it is debatable if on an individual basis, fCP or fEDN levels could be used for clinical follow-up and dietary compliance monitoring. However, prospective studies with larger populations are needed to draw robust conclusions.

## 1. Introduction

Food allergy is defined as an adverse reaction caused by a specific immune response that occurs following exposure to a given food [1]. The immune reaction may be immunoglobulin (Ig)E-mediated, non-IgE-mediated, or mixed. Cow’s milk protein (CMP) is the leading cause of food allergy in infants and children younger than 3 years [2].

Non-IgE-mediated cow’s milk protein allergy (CMPA) may cause enteropathy, proctocolitis, food protein-induced enterocolitis syndrome (FPIES) or minor nonspecific gastrointestinal (GI) manifestations, such as regurgitation, dyspepsia, abdominal pain, or persistent constipation, but also early satiety, anorexia, and food refusal. The severity of reported symptoms is extremely variable.

Due to the non-specific nature of symptoms and the lack of a confirmatory diagnostic test, diagnosis relies on assessing whether symptoms resolve after the suspected allergen has been excluded from the diet, followed by a clinical relapse after reintroduction of the food allergen [1]. Making a correct diagnosis is therefore often a real challenge.

In children with food allergy, fecal calprotectin (fCP) has been reported to be higher compared with healthy controls [3], especially in children with non-IgE-mediated food allergy [4], but in contrast, other studies show no differences [5]. The possible association of early-age high fCP levels as an indicator of the gut immune system in the later development of allergic diseases has also been reported [6]. Moreover, higher levels of fecal eosinophil-derived neurotoxin (fEDN) concentrations have been found in CMPA toddlers compared with controls, although with no statistically significant difference has been found [7].

Literature on the topic is scarce and reports controversial data regarding the clinical value of either of the two markers in young children with gastrointestinal symptoms caused by non-IgE-mediated CMPA. Our aim is to assess the efficiency of fCP and fEDN as diagnostic markers of CMPA, and for monitoring the infants’ response to a CMP-free diet.

## 2. Materials and Methods

### 2.1. Study Population

From January 2015 to December 2018, infants attending the Primary Health Care Center of Bétera and the Pediatric Unit of Casa La Salud Hospital were consecutively invited to participate. We prospectively recruited infants aged from 0 to 9 months, born at term, suspected of non-IgE-mediated CMPA or functional gastrointestinal disorders. The most frequently presenting symptoms were diarrhea, hematochezia, vomiting, or severe constipation with either normal nutritional status or malnutrition, or minor symptoms such as regurgitations and colic with normal nutritional status.

Infants with a diagnosis of CMPA (group 1) according to internationally accepted criteria (1) were put on a CMP-free diet. Stool samples from these infants were taken while the infants were still on a CMP-containing diet (M0); bottle fed babies were given a conventional CM based infant formula (IF) and the breast-fed (BF) children had mothers that were taking CMP in their diet. Additional samples were taken 1 (M1) and 3 months (M3) after starting a CMP-free diet to assess the response to dietetic treatment.

In infants with a diagnosis of mild functional GI disorders (FGID) (group 2) according to Roma IV criteria [8], stool samples were collected only at recruitment (M0).

Additionally, in the same period, we recruited healthy children born at term (group 3) pertaining to families of the hospital staff or patient’s relatives at the Pediatric Department of La Fe Hospital and from the Pediatric Department of La Salud Hospital during regular visits scheduled according to the National Health System protocols. Three stool samples were collected from each infant in the same period as the CMPA children (M0, M1 and M3), with no dietary or medical intervention in this period.

Furthermore, we collected samples from children who presented mild gastrointestinal (GI), upper-respiratory or urinary tract infections (group 4). One or two stool samples were collected from each infant at the acute stage of the disease.

For all children, at recruitment, information on clinical features provided by parents or by the pediatrician (including age, weight and length, sex and vaccines) plus physical examination findings were recorded in a specific case report form (CRF) prior to the stool sample collection. In addition, gestational age, birth weight, mode of delivery (vaginal or cesarean section) and type of feeding (exclusively breast-feeding or formula-feeding) were recorded.

All recruited healthy children met the following inclusion criteria: age 0–9 months, no illnesses or vaccines in the month prior to enrollment, no hospital admissions 3 months prior to enrollment, birth weight appropriate and with no known underlying chronic inflammatory disease. The exclusion criteria were the following: intake of any steroidal or non-steroidal anti-inflammatory drugs, gastric acidity inhibitors, antibiotics or any other drug during the 2 weeks prior to recruitment, or a history of signs or symptoms of infection or gastrointestinal disease (diarrhea, vomiting, hematochezia, and fever).

The present study was approved by the Ethics Committee of La Fe University Hospital. The number of ethical approval was 2014/0157. Written informed consent was obtained from the parents.

### 2.2. Methodology

Parents were provided with a specific plastic screw-capped container and were instructed to collect a small amount of feces from the diapers immediately after defecation. The containers with the stool samples were kept in the fridge at home and then brought to the laboratory no later than 3 days after collection and were then stored at −20 °C until analysis. The protein extraction procedure of the samples, as a preliminary step of the analysis, was performed using the Fecal Sample Preparation Kit^®^ (Roche Diagnostics, Rotkreuz, Switzerland) according to the manufacturer´s instructions.

The fCP and fEDN levels of stool samples were measured in duplicates, by the EliA Calprotectin 2 and ImmunoCAP EDN research assays (Thermo Fisher Scientific, Uppsala, Sweden), respectively. All samples pertaining to one child were analyzed in the same test run.

### 2.3. Statistical Methods

Data were summarized as mean (standard deviation) and median (1st, 3rd quartile) in the case of continuous data, and by absolute frequency (relative frequency) in the case of qualitative data.

To assess differences in fCP and fEDN levels between all groups, a linear regression model was adjusted, including sex and age as confounding variables. To assess the association of fCP and fEDN levels with the CMPA infants and the healthy group over time, two linear mixed effect models with a random intercept were adjusted, including feeding, sex, and age as cofounding variables. We performed a linear mixed model to evaluate fCP evolution over time in G1. The logarithmic transformation of fCP and fEDN was applied to minimize the skewed values. Post hoc Tukey test was performed to assess differences between groups. *p*-values < 0.05 were considered statistically significant. All statistical analyses were performed using R (version 3.5.3, R Foundation for Statistical Computing, Vienna, Austria).

## 3. Results

### 3.1. Baseline Characteristics

**Figure 1:**
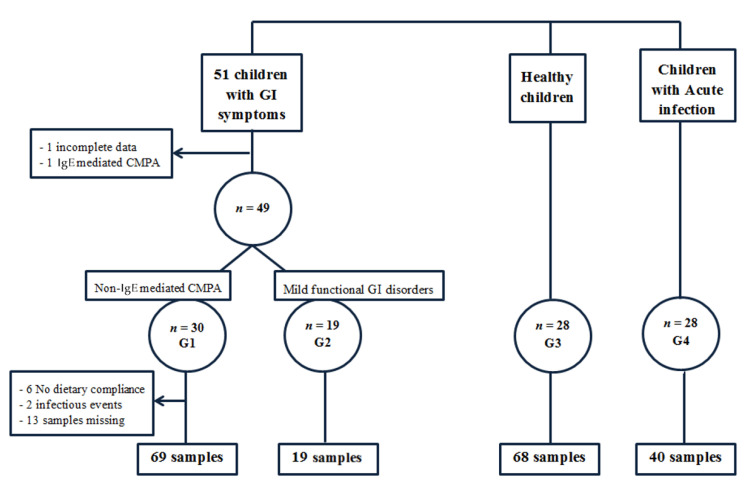
Flow chart of infants included in the study. GI = gastrointestinal; Ig = immunoglobulin, CMPA = cow’s milk protein allergy, G1, G2, G3, G4 = groups.

Initially, 51 infants with GI symptoms were recruited, but 2 were excluded from the study (1 because of incomplete clinical data and 1 because the final diagnosis was IgE-mediated CMPA). Thus, finally, we included 49 infants with GI symptoms (Figure 1), and according to the diagnosis, 2 different groups were considered.

Group 1 (G1) comprised 30 infants (14 girls and 16 boys) with a clinical diagnosis of non-IgE-mediated CMPA, as established by the primary health care pediatrician. The median age at M0 was 1 month and ages at the first and third quartiles were 1 and 2 months, respectively. IgE-mediated allergy was ruled out in all cases. Diagnosis was made on the grounds of strong clinical suspicion, the main GI symptoms identified being colic (*n* = 20), frequent regurgitation (*n* = 15), vomiting (*n* = 7), constipation (*n* = 5), diarrhea (*n* = 12), failure to thrive (*n* = 5), food refusal (*n* = 4) and hematochezia (*n* = 4). In all cases, the diagnosis was confirmed by the pediatrician in charge based on an almost complete resolution of symptoms after a CMP elimination diet, with no other intervention or therapy. In our study, not all children underwent an oral challenge test after 1 month of CMP exclusion because, after clinical improvement, the parents refused it.

At least one (one to three) fecal samples were obtained from each of the 30 children. However, eight samples were discarded because of acute infectious diseases at the time of sampling (two cases) or because of no compliance with the CMP-free diet (six samples). Additionally, 13 follow-up samples were not collected. Finally, 69 samples from 30 children fulfilled the inclusion and exclusion criteria.

Group 2 (G2): 19 infants (10 girls and 9 boys) with mild GI functional disorders, diagnosed according to Roma IV, with no need for pharmacological therapies or nutritional interventions, were included in this group. The main clinical manifestations were colic, food refusal, diarrhea or constipation. The median age was 1 month and the first and third quartiles were 0.75 and 2 months, respectively.

Group 3 (G3) comprised 28 healthy infants as the control group (14 girls and 14 boys). The median age was 1.5 months and first and third quartiles were 1 and 2 months, respectively. Three stool samples were collected from each infant in the same period as the CMPA children (M0, M1 and M3), with no dietary or medical intervention in this period.

Group 4 (G4) comprised children with minor acute GI, upper-respiratory or urinary tract infections. A total of 28 children were included (11 girls and 17 boys), with a median age of 3 months. First and third quartiles were 2 and 4 months, respectively.

### 3.2. Descriptive Results

Descriptive results are summarized in Table 1, Table 2 and Table 3. In patients with non-IgE-mediated CMPA, the median (1st, 3rd quartiles) fCP level before the CMP elimination diet was 564 (219, 694) mg/kg, and in the healthy control group was 199 (126, 545) mg/kg. The median fCP level after the 1 month on the elimination diet in CMPA children was 196 (120.5, 478.4) mg/kg. As regards fEDN level, the median in patients with non-IgE-mediated CMPA before the CMP elimination diet was 4.6 (1.9, 8.7) mg/kg, and in the healthy children group it was 3.7 (1.2, 7.2) mg/kg. The median fEDN level after 1 month on the elimination diet in the CMPA children was 1.5 (0.7, 2.8) mg/kg.

### 3.3. FCP and FEDN Levels at Recruitment in the Different Groups

**Figure 2:**
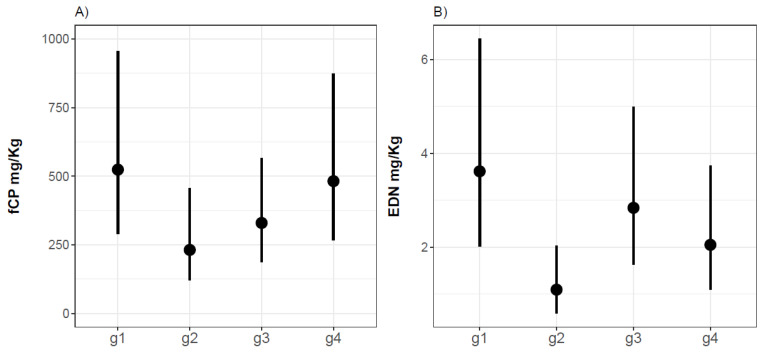
Plot with the distribution of fecal calprotectin (fCP) (**A**) and fecal eosinophil-derived neurotoxin (fEDN) (**B**) concentrations at recruitment for each group: non-IgE-mediated CMPA infants (G1), infants with mild GI functional disorders (G2), healthy infants (G3), and children with infections (G4).

We have compared fCP and fEDN levels from infants with non-IgE-mediated CMPA on a CMP-containing diet (G1), infants with mild GI functional disorders (G2), healthy infants (G3) and children with infections (G4). According to our statistical model results, the levels of fCP in groups 1 and 4 are higher than in group 3 (estimate = 0.467, confidence interval, CI95% (−0.161; 1.095), *p-*value = 0.143) at month 0. Despite this trend, we have not found statistically significant differences between fCP levels, age, or type of feeding between groups (Figure 2).

Regarding fEDN at M0, G2 presented statistically significantly lower fEDN levels (61%) than G3 (estimate = −0.957; *p-*value = 0.007), whereas the fEDN levels in G1 were 46% higher than in healthy children (estimate = 0.238), and those in G4 were lower (28%) than in healthy children (estimate = −0.336); differences between the groups are statistically non-significant according to *p-*values (0.452 and 0.298) (Figure 2).

### 3.4. Biomarkers at Follow-Up in CMPA vs. Healthy Infants

**Figure 3:**
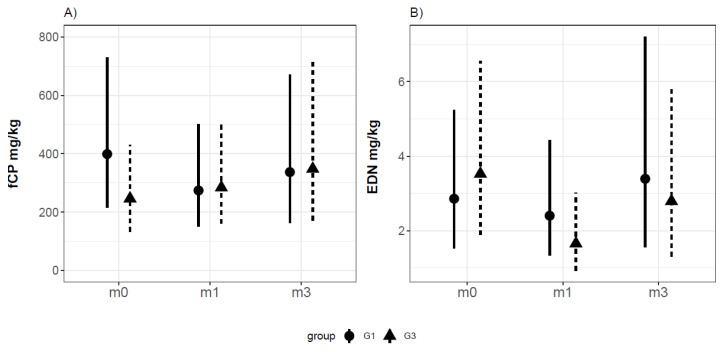
Partial dependence plot of fCP (**A**) and fEDN (**B**) concentrations in samples from non-IgE-mediated CMPA infants (G1) and healthy infants (G3).

At M0, the fCP levels in G1 were 60% higher than those in healthy controls (G3), although they were not statistically significantly (estimate = 0.494, CI95% (−0.101; 1.085), *p-*value = 0.119). Additionally, we did not find statistically significant differences between groups G1 and G3 at M1 (estimate = −0.53, CI95% (−1.24; 0.19), *p-*value = 0.15) nor at M3 (estimate = −0.55, IC95% (−1.35; 0.27), *p-*value = 0.19); no differences were observed between the groups in terms of the fCP trend over time. Figure 3 shows the fCP values for the CMPA infants and the healthy population at inclusion (M0) and after 1 (M1) and 3 (M3) months of follow-up.

Moreover, IF feeding compared to BF (estimate = −0.58, *p*-value = 0.027) in G1 as well as in G3 is related to lower fCP.

The fEDN values did not show differences between groups (estimate = 0.209, CI95% (−0.385; 0.804), *p*-value = 0.506), and no differences were found in children with IF feeding (estimate = −0.028, CI95% (−0.539; 0.485), *p*-value = 0.919) compared to BF. Figure 3 shows the fEDN for the three sampling times in CMPA and healthy infants.

### 3.5. FCP and FEDN Evolution on a CMP-Free Diet

We have compared fCP and fEDN levels before and after starting the CMP exclusion diet in infants with non-IgE-mediated CMPA (group 1). Our data showed that mean the fCP values decreased by 30% from M0 to M1, while the fCP values increased by 37% from M1 to M3. No statistically significant differences were found between the fCP levels corresponding to M0 compared to M1 or M3 (*p*-values 0.184 and 0.899, respectively).

**Figure 4:**
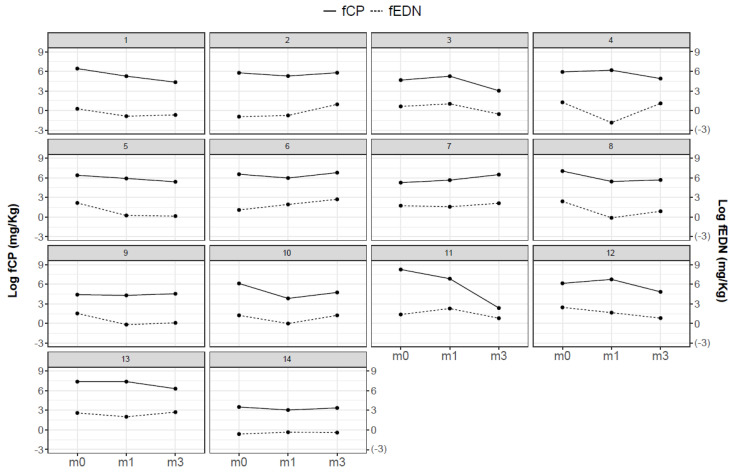
Individual evolution of fCP and fEDN over time in infants of G1 providing 3 samples. The dashed line represents fEDN and the solid line fCP. Variables were log-transformed to improve visualization.

Despite the general trend towards fCP levels decreasing from M0 to M1, there were individuals who did not follow this pattern. We observed that in some cases, the reduction was more evident after 3 months on dietary restriction. We show in Figure 4 the evolution of the fCP and fEDN levels for the infants who correctly collected the three serial samples at M0, M1 and M3. Although different patterns were observed, the two biomarkers fCP and fEDN follow similar trends on an individual basis.

Moreover, the evolution in fCP levels from M0 to M1 was similar in children with constipation and diarrhea (CI95% (−1.69; 0.72)).

In analyzing fEDN, we found lower fEDN levels (52%) at M1 than at M0 (estimate = −0.745, CI95% (−1.23, −0.253), *p-*value = 0.005), but no statistically significant differences between M3 and M0 (*p-*value = 0.728) according to *p-*values.

## 4. Discussion

According to our study results, the variability in fCP and fEDN levels in young infants is high, as shown by the wide standard deviation found for both parameters; however, CMPA infants had higher fCP and fEDN values at diagnosis (normal diet) compared to healthy ones, although the differences do not reach statistical significance.

Several published studies obtained higher levels of fCP in non-IgE CMPA groups compared to control groups. Beşer et al. [4] found highly statistically significant fCP levels before the commencement of a CMP elimination diet in a subgroup of 8 non-IgE CMPA infants (median age 2.8 months) compared to 39 control infants (median age 11.5 months); however, the age difference between the two groups could influence these results, since age has a negative association with fCP levels [9]. Trillo et al. [10] observed differences between 40 non-IgE CMPA infants (mean age 3.7 months) and 30 control patients (mean age 3.8 months), obtaining a statistically significant relationship between high fCP levels and non-IgE CMPA. The authors also found statistically significant associations between elevated mean fCP values and rectal bleeding in 10 infants with hematochezia. Baldassarre et al. [11] found statistically significantly higher fCP levels (*p* < 0.0001) in 30 infants (mean age 4 months), all with hematochezia (mixed IgE and non-IgE-mediated CMPA), as compared with a healthy group (mean age 4 months).

In our study, only 4 out of 30 children with CMPA had hematochezia, which, together with the differences in the study design, could explain the fact that although we observed differences in fCP levels in the CMPA group compared to controls, we did not find statistical significance. In further studies, the performance of fecal occult blood tests could eventually be used to identify proctocolitis patients and evaluate them as a specific clinical group.

Our results show that after 1 month on an elimination diet, there is a tendency towards a reduction in fCP and fEDN values in the CMPA group, which was not observed in the healthy population. Although it is not statistically significant, this trend could be related to a decrease in the inflammatory pattern secondary to dietary exclusion. A recent study [5] showed that the fCP levels in 17 infants aged up to 2 years, with non-IgE-mediated CMPA and on a CMP-free diet for at least 6 months, were not different compared to 10 age-matched healthy controls. However, no data on fCP are reported before dietary exclusion, and the mean age of the study population is not comparable to our infants’ ages.

Previously published studies also refer to decreasing levels of fCP after starting the elimination diet. In 29 non-IgE CMA colitis infants (mean age 4 months), Ataee et al. [12] found that the fCP levels decreased 2 and 6 weeks after starting the diet, but there was no statistically significant difference. However, Beser et al. [4] found that the fCP values were significantly lower after the CMP elimination diet in the non-IgE CMPA group. In the study of Trillo et al. [10], there was a statistically significant relationship between high fCP levels and non-IgE CMPA, not only at diagnosis, but also 1 and 3 months after the elimination diet. Baldassarre found a 50% reduction in fCP in the CMPA group, after 4 weeks of the elimination diet, but the fCP was still significantly higher than in controls [11]; remarkably, a significant reduction in fCP was also observed in the control group. Merras-Salmio et al. [13] assessed fCP during elimination diets, and after double-blind placebo-controlled food challenges with 55 infants and young children (median 8 months), the authors reported that in children with non-IgE CMPA, there were no differences in fCP concentrations from those measured under CMP-free diet and challenge.

Although in our population, the levels of fCP decreased by 30% from M0 to M1 in CMPA children, there was a tendency towards a slight mean increase in fCP and fEDN at M3. This observation, noticed only in some infants, is difficult to explain, as close clinical follow-up confirmed that patients were compliant with the CMP-free diet and that no clinical symptoms were reported at that time. Interestingly, the higher values observed at M3 in CMPA children are similar to the ones observed for the healthy children after the same follow-up time and for the same age range (4–6 months). Thus, the potential impact of age-related factors needs to be considered, and among them the feeding pattern, i.e., the start of the complementary feeding and switch from mixed formula (MF)/BF to IF feeding. Further studies need to be carried out to analyze the impact of the feeding pattern on the fCP normal values, as well as other factors such as vaccinations received.

The published studies on fCP in non-IgE-mediated CMPA children are heterogeneous with respect to different clinical pictures, analysis methods, sample sizes, ages of children, sampling times after CMP exclusion, the inclusion or not of a control group, and the mixing of non-IgE and IgE-mediated CMPA patients. In a recent review by Xiong et al. [14] including thirteen studies with IgE-mediated and non-IgE-mediated CMPA children, the authors concluded that the available evidence was not sufficient to confirm the utilization of fCP, neither for diagnosis nor for the monitoring of CMPA.

In infants with mild infections, in the present study we observed higher fCP values, although these were not statistically significant when compared with controls. Minor infections, even non-gastrointestinal ones, could probably also have an impact on fCP levels [15]. This fact could result in uncertain clinical interpretations if fCP is used as a biomarker for CMPA diagnosis in infants, as mild infections are frequent at this young age.

Studies regarding fEDN in non-IgE-mediated CMPA children are scarce. A prospective study [7] measured different fecal markers in 11 CMPA (8 non-IgE-mediated) and in 14 control patients (mean age 6 months). Although not statistically significant, higher fEDN levels in CMPA patients were observed compared to controls; they concluded that the measurement of fEDN is promising in this population. This is consistent with our results showing a reduction in fEDN after 1 month on a CMP elimination diet. Two additional clinical cases have been reported [16,17], in which fEDN levels increased significantly after an oral challenge. Recently, a prospective study in infants 1–12 months of age found that median fEDN and fCP levels were significantly higher in 27 non-IgE-mediated food allergy (85% CMPA) children with hematochezia compared to a control group with functional disorders [18].

One limitation of our study is that, due to the inter-individual variability in the fCP and fEDN levels and the heterogeneity of clinical manifestations, the results present wide confidence intervals, indicating that more in-depth studies with larger populations (allowing subgrouping according to different clinical pictures) will be necessary to draw more precise conclusions. The need to exclude eight samples from healthy children because of minor infections reflects the frequency of mild infections in young infants, which may inadvertently influence the results. Tests of serum IgE specific to CMP or a skin prick test of IgE were not performed for all children, as they had no warning signs of IgE-mediated allergy.

According to the above results, especially due to the inherently high variability of fCP and fEDN levels at this early age [9], it seems that neither fCP nor fEDN levels are helpful to discriminate between healthy infants and those with signs or symptoms related to CMPA. Additionally, it is debatable whether fCP or fEDN levels on an individual basis could be used for clinical follow-up and dietary compliance monitoring, taking into account discrepancies in the individual behaviors of these markers after a CMP-free diet is started. However, considering trends observed in both fCP and fEDN in our research, prospective studies with larger populations are needed to draw robust conclusions in non-IgE CMPA infants. Future studies should thus address additional factors that can affect levels of fCP and fEDN, such as age, blood in stool and /or concomitant infections, complementary feeding patterns, or vaccinations.

## Figures and Tables

**Table 1:** Summary of fCP and fEDN in non-IgE-mediated CMPA children (G1) before (M0) and after one (M1) and 3 months (M3) of the CMP elimination diet.

Variable	G1 M0 (*n* = 25)	G1 M1 (*n* = 26)	G1 M3 (*n* = 18)
	Mean (SD)/*n* (%) Median (1st, 3rd Q.)	Mean (SD)/*n* (%) Median (1st, 3rd Q.)	Mean (SD)/*n* (%) Median (1st, 3rd Q.)
Age (months)	1.68 (1.32)	2.5 (1.45)	4.39 (1.65)
	1 (1, 2)	2 (2, 3)	4 (3.25, 5)
fCP (mg/kg)	726.06 (844.79)	347.6 (353.35)	268.44 (244.03)
	564.25 (219.03, 693.83)	196.25 (120.53, 478.36)	203.05 (98.71, 352.83)
fEDN (mg/kg)	5.79 (4.33)	2.85 (3.5)	4.36 (5.2)
	4.64 (1.91, 8.65)	1.49 (0.72, 2.82)	2.34 (1.12, 3.34)
	**Mean (SD)/*n* (%)**	**Mean (SD)/*n* (%)**	**Mean (SD)/*n* (%)**
Feeding			
BF	13 (52%)	11 (42.31%)	5 (27.78%)
IF	7 (28%)	10 (38.46%)	8 (44.44%)
MF	5 (20%)	5 (19.23%)	5 (27.78%)
Sex			
F	11 (44%)	11 (42.31%)	8 (44.44%)
M	14 (56%)	15 (57.69%)	10 (55.56%)
CMP ED	0 (0%)	26 (100%)	18 (100%)

BF = breast-fed; IF = infant formula; MF = mixed feeding; F = female; M = male; CMP = cow´s milk protein; CMP ED = CMP elimination diet; CMPA = cow’s milk protein allergy; SD = standard deviation; fCP = fecal calprotectin; fEDN = fecal eosinophil-derived neurotoxin.

**Table 2:** Summary of fCP and fEDN in healthy children (G3).

Variable	G3 M0 (*n* = 28)	G3 M1 (*n* = 23)	G3 M3 (*n* = 17)
	Mean (SD)/*n* (%) Median (1st, 3rd Q.)	Mean (SD)/*n* (%) Median (1st, 3rd Q.)	Mean (SD)/*n* (%) Median (1st, 3rd Q.)
Age (months)	1.67 (1.01) 1.5 (1, 2)	2.7 (1.15) 3 (2, 3)	4.65 (1.46) 4 (4, 6)
fCP (mg/kg)	448.98 (538.63) 199.43 (126.32, 545)	587.17 (1165.59) 228.82 (73.62, 472.85)	379.27 (655.5) 100.66 (64.05, 318.58)
fEDN (mg/kg)	5.66 (7.1) 3.74 (1.23, 7.23)	4.4 (4.98) 2.15 (1.1, 5.46)	6.48 (10.67) 2.48 (1.51, 3.42)
	**Mean (SD)/*n* (%)**	**Mean (SD)/*n* (%)**	**Mean (SD)/*n* (%)**
Feeding			
BF	14 (50%)	12 (52.17%)	6 (37.5%)
IF	11 (39.29%)	10 (43.48%)	6 (37.5%)
MF	3 (10.71%)	1 (4.35%)	4 (25%)
Sex			
F	13 (46.43%)	11 (47.83%)	8 (47.06%)
M	15 (53.57%)	12 (52.17%)	9 (52.94%)
CMP CD	28 (100%)	23 (100%)	17 (100%)

BF = breast-fed; IF = infant formula; MF = mixed feeding; F = female; M = male; CMP CD = CMP-containing diet.

**Table 3:** Description of G1, G2, G3 and G4; and mean, standard deviation, median and first and third quartile levels of fCP and fEDN from infants with CMP consumption in their diet (sample M0) from the 4 groups.

Variable.	G1 M0 (*n* = 25)	G2 M0 (*n* = 19)	G3 M0 (*n* = 28)	G4 M0 (*n* = 28)
	Mean (SD)/*n* (%) Median (1st, 3rd Q.)	Mean (SD)/*n* (%) Median (1st, 3rd Q.)	Mean (SD)/*n* (%) Median (1st, 3rd Q.)	Mean (SD)/*n* (%) Median (1st, 3rd Q.)
Age (months)	1.68 (1.32) 1 (1, 2)	2.19 (2.37) 1 (0.75, 2)	1.67 (1.01) 1.5 (1, 2)	3.01 (1.66) 3 (2, 4)
fCP (mg/kg)	726.06 (844.79) 564.25 (219.03, 693.83)	234.59 (206.24) 206.79 (101.58, 300.68)	448.98 (538.63) 199.43 (126.32, 545)	660.57 (822.46) 340.85 (141.25, 789.09)
fEDN (mg/kg)	5.79 (4.33) 4.64 (1.91, 8.65)	2.19 (2.78) 1.25 (0.62, 2.17)	5.66 (7.1) 3.74 (1.23, 7.23)	3.97 (4.5) 2.21 (0.91, 5.25)
	**Mean (SD)/*n* (%)**	**Mean (SD)/*n* (%)**	**Mean (SD)/*n* (%)**	**Mean (SD)/*n* (%)**
Feeding				
BF	13 (52%)	6 (31.58%)	14 (50%)	8 (28.57%)
IF	7 (28%)	8 (42.11%)	11 (39.29%)	17 (60.71%)
MF	5 (20%)	5 (26.32%)	3 (10.71%)	3 (10.71%)
Sex				
F	11 (44%)	10 (52.63%)	14 (50%)	11 (39.29%)
M	14 (56%)	9 (47.37%)	14 (50%)	17 (60.71%)
CMP CD	25 (100%)	19 (100%)	28 (100%)	28 (100%)

BF = breast-fed; IF = infant formula; MF = mixed feeding; F = female; M = male; CMP CD = CMP-containing diet.

## Data Availability

Datasets from this study are available from the corresponding author on reasonable request.
